# Probing the spectral density of the surface electromagnetic fields through scattering of waveguide photons

**DOI:** 10.1038/srep21673

**Published:** 2016-02-10

**Authors:** Guang-Yin Chen

**Affiliations:** 1 Department of Physics, National Chung Hsing University, Taichung 402, Taiwan

## Abstract

The spectral density of the metal-surface electromagnetic fields will be strongly modified in the presence of a closely-spaced quantum emitter. In this work, we propose a feasible way to probe the changes of the spectral density through the scattering of the waveguide photon incident on the quantum emitter. The variances of the lineshape in the transmission spectra indicate the coherent interaction between the emitter and the pseudomode resulting from all the surface electromagnetic modes. We further investigate the quantum coherence between the emitter and the pseudomode of the metal-dielectric interface.

Surface-plasmon polariton (SPP), an electromagnetic (EM) excitation existing on the surface of the metals^1^,^2^, can be excited by the external fields. Resulting from its strong interaction with the quantum emitter (QE), significant enhancement of the atomic or excitonic decay rate has been observed^1^,^2^,^3^,^4^,^5^,^6^,^7^,^8^,^9^. With strong analogies to light propagation in conventional dielectric components^10^,^11^, SPP has been applied to achieve the subwavelength waveguiding below the diffraction limit, the bipartite quantum entanglement^12^,^13^, and to miniaturize existing photonic circuits^14^. The strong coupling between the QE and surface plasmon fields also enables the system to act like a lossy optical cavity, namely, the interaction can be coherent^5^,^7^.

Recently, it has been reported^15^ that with small enough separation between the quantum emitter (QE) and the metal-dielectric interface, the spectra density of the system changes from smooth to Lorentzian, leading to the reversible population dynamics between the quantum emitter and the metal surface. However, it might not be easy to observe this reversible dynamics by measuring the population. In this work, we propose a feasible way to indirectly observe the reversibility in population through the scattering of the waveguide photon incident on the quantum emitter. The variances of the transmission lineshapes reveal the existence of the coherent coupling between the quantum emitter and the dominant electromagnetic modes of the metal surface. This proposal can be further applied to detect the defects in the system with excitonic coherent couplings such as the excitation transfer in the photosynthetic complex^16^.

## Results

**Figure 1:**
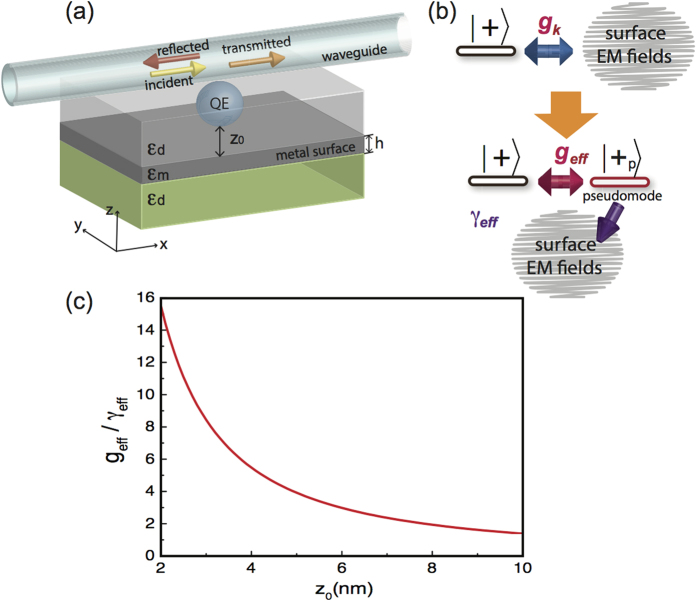
Schematic diagram of the system. (**a**) Schematic diagram of a quantum emitter coupled to dielectric-metal interface. A single waveguide-photon incident on the quantum emiiter enables us to detect the coherent interaction between the quantum emitter and the metal surface. In this work, we consider silver thin film with thickness *h* = 5 nm to be the metal surface. We have assumed the silver film is embedded into a dielectric material with 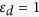. Here 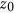 is the separation between the quantum emitter and the silver surface, and the permittivity of the silver film 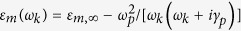 with the plasma frequency 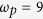 eV, 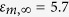, and the Ohmic losses 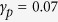 eV. (**b**) An illustration of the model. When the separation 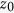 is small enough, the spectra density of the system becomes Lorentzian. The system can then be regarded as a quantum emitter coherently coupled to a pseudomode with dissipative Ohmic losses. (**c**) The ratio of the effective coupling strength 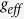 and the decay rate into Ohmic losses 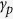 as a function of the separation 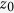.

We consider a two level QE positioned close to a two-dimensional metal-dielectric interface as depicted in Fig. 1(a). The QE is coupled electromagnetically to the SP modes on the metal surface. The Hamiltonian of this QE-metal film system can then be written within the rotating-wave approximation^17^ as:


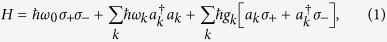


where 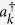 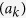 is the creation (annihilation) operator of the *k*-mode surface-EM-filed, 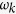 is the frequency of the *k*-mode surface-EM-field, and 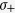 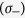 is the raising (lowering) operator for the QE. Here, 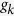 describes the coupling strength between the QE and the *k*-mode surface-EM-field, and 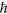 is set to be unity throughout the paper.

A recent research^15^ studied the spectra density which comprises information about the density of the surface EM-fields, and also the QE-field coupling. The results reveal that when the separation 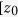, as seen in Fig. 1(a)] between the QE and the metal surface decreases to a small distance (≤10 nm), the spectra density 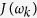 can change from smooth to the Lorentzian distribution,


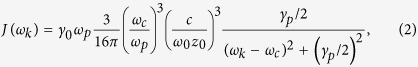


where, 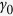 is the spontaneous decay rate of the QE into free space. In this work, we consider the silver thin film with thickness *h*=5 nm to be the metal surface. Therefore, 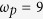 eV (for silver) is the plasma frequency, and 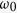 denotes the energy spacing of the QE. Here, *c* is the speed of light, 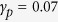 eV (for silver) denotes the field-damping rate of the surface-EM-field into Ohmic losses, and 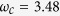 eV (for silver thin film) is the main peak of the Lorentzian function.

As a result, the Lorentzian spectra density of the system allows us to map the system to the excited state of QE coherently coupled with a strength 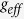 to a pesudomode^15^,^18^ with a Markovian dissipation 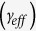 as depicted in Fig. 1(b). The dynamical evolution of the QE is govern by the master equation^15^,


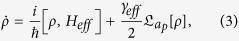


with 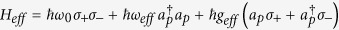 describing the energy of the QE, the pseudomode 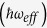 and the coherent interaction between them with strength 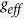. Here 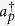 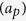 is the creation (annihilation) operator of the pseudomode. In Eq. (3), 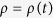 is the density matrix of the total system. By taking the notation 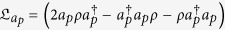, the Lindblad term, 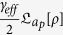 describes the surface-EM-field damping with rate 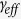 into Ohmic loss.

According to Eq. (3), the population shows reversible dynamics between the QE and the pseudomode which represents all the electromagnetic modes of the metal surface. Since the pseudomode is originated from the Lorentzian spectra density [Eq. (2)] of the system, its energy 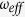 is exactly the main peak 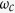. The effective loss 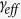 of the pesudomode corresponding to the width of the Lorentzian function is the Ohmic loss 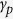, while the coherent coupling strength 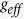 can be given by^15^


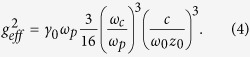


In Eq. (4), we can see that since 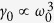, the dependence on 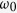 cancels, the effective coherent coupling strength then depends mainly on the separation 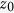. In Fig. 1(c), we plot the coupling 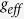 as a function of the separation, and as can be seen, it decreases with growing separation 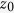, which coincides with its near-field nature^1^. Notice that when the separation 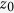 increases, the Lorentzian spectra becomes flatter, and the coherent interaction fades out accordingly^15^.

Experimentally, to observe the reversible dynamics of the system, one needs to perform the time-resolved measurements to the population evolution of the QE. However, the reversible dynamics inevitably suffers the dissipations such as the Ohmic losses. It might not be easy to observe the dynamics in practice.

Here, we propose a feasible way to detect the existence of the coherent interaction between the QE and the pseudomode through scattering of the waveguide-photon fields. We consider a waveguide coupled to the QE as shown in Fig. 1(a). A single waveguide-photon injected from the left is coherently scattered by the QE. After performing the rotating-wave approximation^17^, the total Hamiltonian of the system with the additional probing waveguide-photon fields becomes,


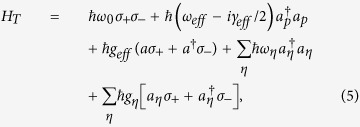


where 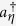 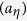 is the creation (annihilation) operator of the *η*-mode waveguide-photon, 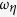 is the frequency of the waveguide-photon, and 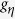 describes the coupling strength between the QE and the *η*-mode waveguide-photon, which leads to the decay of the QE into the waveguide. Note that, here the QE is coupled to both the waveguide-photon-fields and the surface-EM-fields. However, since the surface-EM-field decays exponentially with distance from the surface^1^,^2^, the interaction between the surface-EM-fields and the waveguide is extremely small (see detailed discussion in the experimental realization part), and cannot affect the coupling between the emitter and the waveguide. The coupling strength 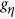 can therefore be treated as a perturbation to the original QE-metal film system and can be further assumed to be frequency-independent, this assumption is equivalent to the Markov approximation^19^,^20^. Because we are only interested in the way the system behaves in experiments, it is sufficient to include the dissipative channels in Eq. (3) via introducing the non-Hermitian term 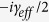 in the total Hamiltonian and the “quantum jump”^21^ term can be neglected.

The scattering eigenstate state of the above combined system can be written as^5^,^22^:


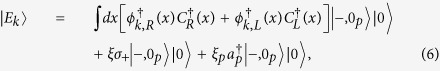


where 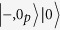 describes that the QE is in the ground state with no excitation in the pseudomode and the photon field state, while 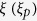 is the probability amplitude that the QE (pseudomode) absorbs the excitation. We also assume that the field is incident from the left of the waveguide, 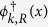 and 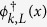 therefore take the form,


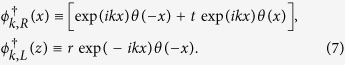


Here, *t* and *r* are the transmission and reflection amplitude, respectively, and 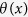 is the unit step function. The total Hamiltonian [Eq. (5)] can be further transformed^22^,^23^,^24^ into real-space representation, 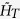, and applied to the scattering eigenstate [Eq. (6)]. The transmission spectrum 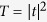 and the probability amplitudes *ξ*, *ξ*_*p*_ can then be obtained by solving the eigenvalue equation 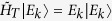.

**Figure 2:**
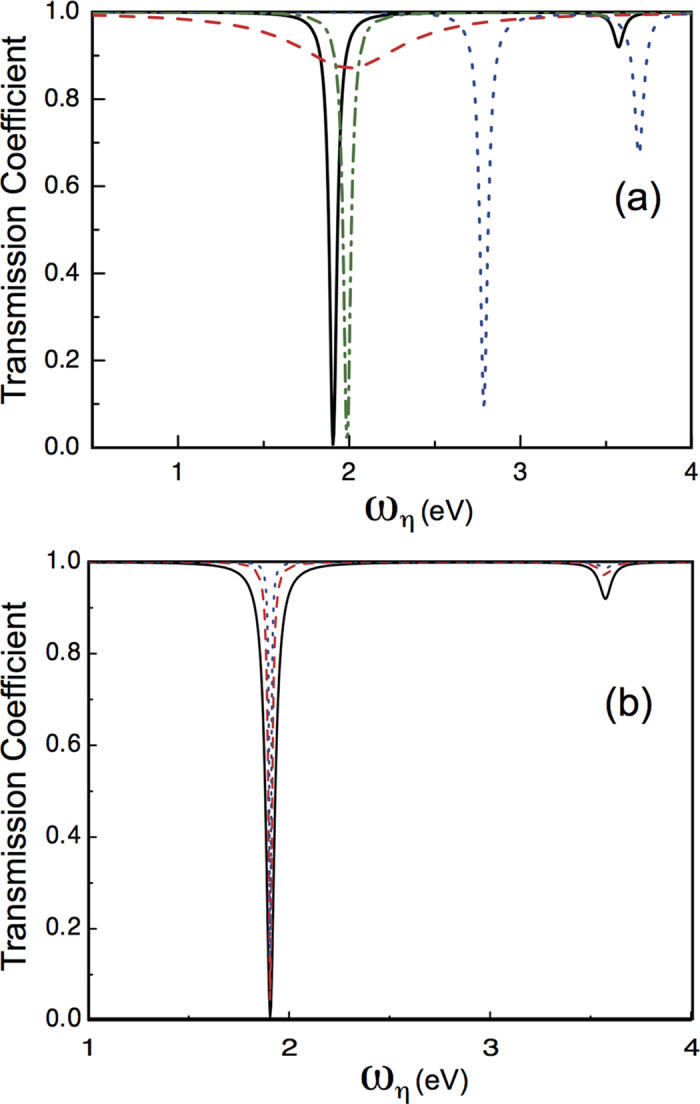
The scattering spectra of the incident waveguide-photon. (**a**) The transmission coefficient *T* as a function of the energy of the incident waveguide-photon. The black-solid curve takes the parameters: 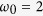 eV, 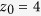 nm, and 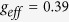 eV. The red-dashed curve is for 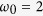 eV, 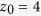 nm, 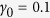 meV, and 
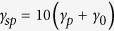
. The green-dotted-dashed curve is for 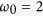 eV, 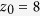 nm, and 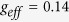 eV. The blue-dotted curve is for 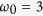 eV, 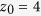 nm, and 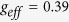 eV. Notice that in plotting (**a**), the coupling strength 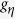 between the waveguide-photon and the quantum emitter is fixed to be 50 meV. (**b**) Comparisons of the transmission spectra for different coupling strength 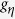. The black-solid curve adopts all the parameters of the black-solid curve in (**a**), the red-dashed uses 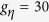 meV, and the blue-dotted curve uses 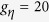 meV.

In Fig. 2(a), we plot the transmission coefficient 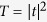 for different energy spacings of the QE, QE-metal separations, and the coupling strengths 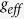 as functions of the energy of the incident waveguide-photon. If there is no coherent interaction 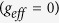, namely, the spectra density of the surface EM-fields is smooth without significant signatures of resonant peaks, the QE then interacts with the continuum of SPP modes [see Eq. (1)] with equal coupling strength 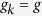, leading to a strong decay rate^5^ 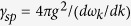 into SPPs. This rate can be included in the QE Hamiltonian with an additional non-Hermitian term 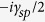. Here the SPP modes, not a pseudomode, plays the role of a dissipative Markovian environment. In the QE-SP system, due to the relative strong coupling, the rate 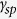 can be large compared with 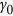 and 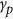, we set^1^,^5^,^25^ 
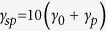
 in this work. As seen in Fig. 2(a), the profile of the transmission coefficient *T* (red-dashed curve, with 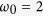 eV, 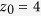 nm) is just the dissipative single particle transmission lineshape^5^,^12^. However, when the spectral density of the surface-EM-fields is Lorentzian, the QE-pseudomode coherent coupling 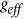 exists (thus, 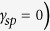, the profile of *T* (black-solid curve, with 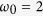 eV, 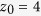 nm, and 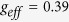 eV) significantly changes to two-dip lineshape. We therefore can precisely detect the existence of the coherent interaction between the QE and the pesudomode.

The green-dotted-dashed curve (with 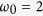 eV, 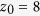 nm, and 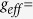
0.14 eV) in Fig. 2(a) shows that for larger separation 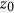, 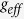 decreases as shown in Fig. 1(c), leading to a remarkably different profile comparing with the black-solid curve. In order to compare the profiles of *T* with different energy spacings of the QE, we also plot the blue-dotted curve (with 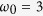 eV, 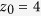 nm, and 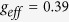 eV). As can be seen, it has a different zero point of *T* from the case of 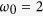 eV. These results indicate that through the scattering of the waveguide-photon, we can detect not only the existence of the coherent interaction, but the varies of the coupling, and even the effect of different energy spacings of the QE from the transmission spectra.

In plotting Fig. 2(a), we assume the coupling between the waveguide-photon and the QE is 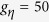 meV. However, this coupling 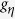 in practice varies with the QE-waveguide separation^26^, we therefore plot the transmission coefficient *T* with different 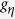 in Fig. 2(b). As shown, although the lineshape becomes sharper when reducing the strength of 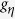, the profile can still be distinguished. This means that even with small 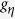, we can still detect the existence of the coherent interaction 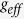.

## Discussion

**Figure 3:**
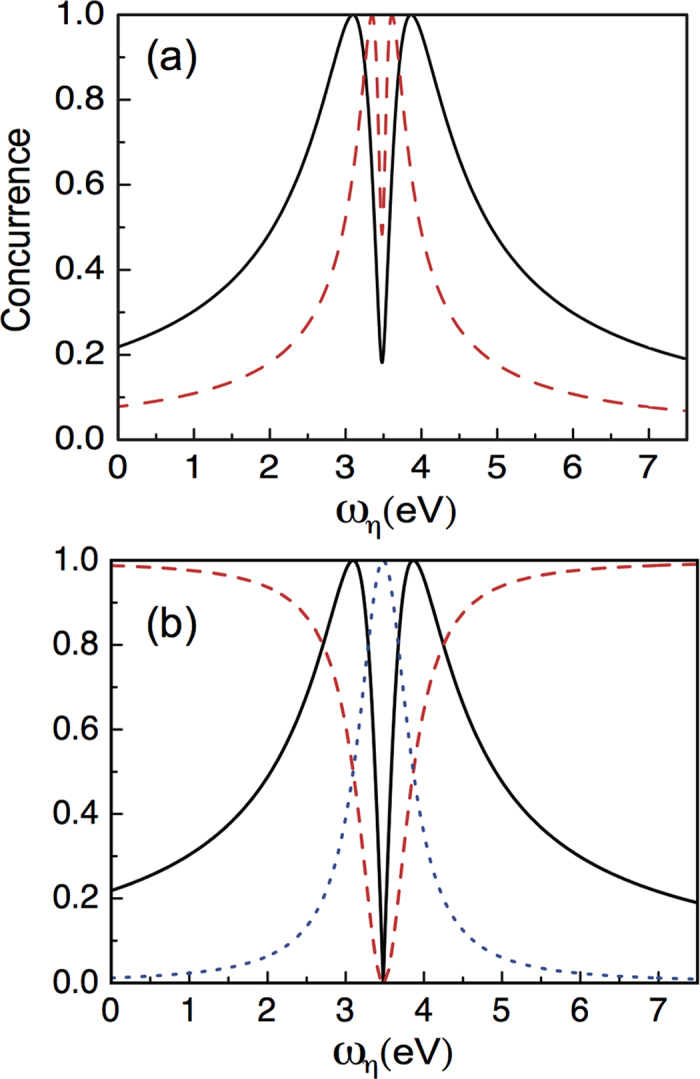
The quantum coherence between the quantum emitter and the pseudomode. (**a**) The concurrence of the quantum emitter and the pseudomode as a function of the energy of the incident waveguide-photon. The black-solid curve takes the parameters 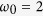 eV, 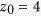 nm, and 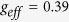 eV. The red-dashed curve takes: 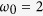 eV, 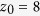 nm, and 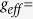
0.14 eV. (**b**) The black-solid curve takes all the parameters of the black-solid curve in (**a**) but without dissipations 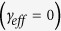. The red-dashed (blue-dotted) curve is the modulus of the probability amplitude 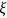 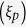. In plotting this figure, the coupling strength 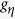 is set to be 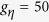 meV.

Since the pseudomode has the coherent interaction with the QE, it will be interesting to study the quantum coherence between the QE and the pseudomode. We calculate the concurrence^27^ which quantifies the degree of the bipartite entanglement and also shows the behavior of the quantum coherence. In our scattering approach, after tracing out the microwave-photon fields, the reduced density matrix of the bipartite state is a pure state, and the concurrence simply takes the form 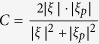. In Fig. 3(a), we plot the concurrence as black-solid (red-dashed) curve for parameters adopted from the black-solid (green-dotted-dashed) curve in Fig. 2(a). As can be seen in Fig. 3(a), the concurrence is non-zero positive, indicating that there exists quantum coherence between the QE and the pseudomode. One can also see that there is a dip in the concurrence lineshape, and when 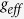 decreases, the dip shrinks. In order to see more insights about the behavior of the concurrence, in Fig. 3(b) we plot the black-solid curve in (a) again without the dissipations, meanwhile, we also plot the normalized 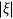 (red-dashed) and 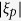 (blue-dotted). The dips occurs when the energy of the incident waveguide-photon 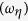 is resonant with the energy of the pseudomode 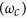. This is because in our scattering approach, the QE state and the pseudomode are within a different subspace from the field states. When the excitation is in the QE, it transfers to the pseudomode via the coherent interaction, the excitation therefore completely transfers to the pseudomode when 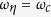. This also explains why the dip shrinks when the coherent coupling 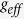 becomes smaller.

For the experimental realizations, high QE-waveguide coupling strength can be achieved with dielectric waveguides such as photonic crystal waveguides^28^ and dielectric slot waveguides^29^. However, in order not to seriously affect the spatial structure of the surface-EM-fields, we suggest to utilize the plasmonic nanowire^30^ as the probing waveguide, and the II–VI colloidal quantum dots (e.g., CdSe/ZnS quantum dots with exciton energy around 2–2.5 eV) placed close to a silver thin-film to form the QE-metal film system. Due to the nature that being a near field, the intensity of the surface-EM fields decays exponentially away from the surface^1^,^2^. In this work, we set the QE-waveguide coupling 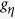 to be a few tens of meV, which is small compared with the QE-metal film coupling 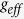 (∼a few hundreds of meV). Figure 1(c) and Eq. (4) gave us a rough estimation that to achieve this magnitude of the coupling strength, the plasmonics metal-nanowire waveguide is about 30 nm away from the QE. Given the physical size of the II–VI colloidal quantum dot (∼10 nm) and the QE-metal film separation (∼5 nm), the plasmonic waveguide is actually about 50 nm away from the metal film. With this separation, the intensity of the surface-EM fields decays to 6 orders smaller^15^. It therefore can only very slightly affect the plasmonic nanowire waveguide, and similarly, the fields of the waveguide can only slightly affect the metal thin film. This is the reason we treat the waveguide fields and the surface-EM fields as independent fields, and QE-waveguide coupling 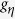 as a perturbation to the original QE- metal film system. The plasmonic metal-nanowire therefore plays a role probing the reversible dynamics in the QE-metal film system.

Summarizing, we propose that through the scattering of the waveguide-photon incident on a quantum emitter, one could precisely measure the coherent interaction leading to reversible dynamics in population between the quantum emitter and the dielectric-metal interface. The behavior of the quantum coherence between the quantum emitter and the metal surface has been also studied by calculating the bipartite entanglement.

## Additional Information

**How to cite this article**: Chen, G.-Y. Probing the spectral density of the surface electromagnetic fields through scattering of waveguide photons. *Sci. Rep.* **6**, 21673; doi: 10.1038/srep21673 (2016).
